# Under pressure - a sensor-based analysis of simulated prehospital pressure dressing application for hemorrhage management

**DOI:** 10.1007/s00068-026-03247-9

**Published:** 2026-06-15

**Authors:** Vesta Brauckmann, Jonathan Menzel, Bastian Welke, Sebastian Decker, Christian Macke

**Affiliations:** 1 Department of Trauma Surgery, Hannover Medical School, Carl- Neuberg-Straße 1, D-30625 Hannover, Germany; 2 Laboratory for Biomechanics and Biomaterials, Department of Orthopedic Surgery, Hannover Medical School, Anna-von-Borries-Str. 1- 7, 30625 Hannover, Germany

**Keywords:** Prehospital emergency care, Emergency medical services, Trauma, Emergency medicine, Hemorrhage control, Pressure dressing, External bleeding, Bandage, Pressure measurement

## Abstract

**Background:**

The concept of “x-ABCDE”, with “X” representing exsanguinating hemorrhage, highlights the priority of immediate bleeding control in trauma care. Despite being a fundamental intervention, pressure dressing techniques lack standardization. Studies show high variability in achieved pressures, often below therapeutic targets or exceeding harmful levels, with no correlation between subjective estimates and objective measurements.

**Objectives:**

To objectively assess current practices of pressure bandage application and identify influencing factors including material type, technique, and provider experience.

**Methods:**

This prospective, single-center, simulation-based observational study was conducted from August 2024 to January 2025 at a Level I Trauma Center. Emergency medical providers applied pressure dressings according to personal preference using available materials. Pressure distribution was measured via two calibrated capacitive pressure sensors (61 measurement fields). Analysis included correlation tests, group comparisons, and multiple regression.

**Results:**

A total of 124 emergency medical providers (75% male; 36% paramedics, 44% emergency medical technicians, 13% trainees, 7% emergency physicians) completed pressure dressing applications, with 116 datasets included in final analysis. Mean maximum pressure was 169.35 ± 84.33 mmHg (range 50–412 mmHg) with application duration of 51.20 ± 18.14 s. Emergency physicians generated significantly higher pressures than non-physician groups (*p* < 0.001). The short-tug technique was the strongest predictor of maximum pressure (*p* < 0.001), followed by bandage material. Israeli bandages achieved highest pressures (205.40 ± 98.12 mmHg), followed by medium-stretch (171.68 ± 75.03 mmHg) and elastic fixation bandages (135.50 ± 58.63 mmHg). Excessive pressures > 250 mmHg occurred in 28% of Israeli bandage applications versus 11.1% for medium-stretch and 1.6% for elastic bandages. Pressure distribution showed volar focusing with highest values measured by the smaller sensor in the central area, best achieved through medium-stretch bandages. No correlation was found between subjective self-assessment and objective pressure measurements (*p* = 0.541).

**Conclusions:**

Pressure dressing application varies significantly by material, technique, and experience. While the short-tug technique and Israeli bandages achieved highest peak pressures, medium-stretch bandages demonstrated the most therapeutically consistent pressure distribution, with pronounced target pressure focusing and the lowest rate of excessive pressures within the present observational simulation setting. Lack of correlation between self-assessment and objective performance highlights the need for standardized training protocols and evidence-based technique guidelines.

**Supplementary Information:**

The online version contains supplementary material available at 10.1007/s00068-026-03247-9.

## Introduction

Uncontrolled hemorrhage represents one of the leading potentially preventable causes of death following trauma and accounts for approximately one-third of all prehospital trauma fatalities [1–11]. Open extremity injuries in particular can lead to exsanguination within minutes, making early control of external bleeding crucial to increase survival probability [1]. This urgency is reflected in the concept of the “Platinum Ten Minutes”, which demands that prehospital measures for stopping active bleeding be initiated within the first ten minutes [12]. Current national and international trauma algorithms, including PHTLS (Prehospital Trauma Life Support), follow the x-ABCDE scheme - with the prefixed “x” designating exsanguinating bleeding as the priority intervention, addressed even before “A” airway management [5, 13, 14]. 

For the control of external hemorrhage, a graduated approach is considered by emergency medical personnel with different techniques at their disposal. Manual compression is considered the immediate first measure, followed by pressure dressing application as the standard intervention. In this approach tourniquets are reserved as the final option, when other measures prove insufficient [2, 5, 15, 16]. Among modern pressure dressing systems, the Israeli Emergency Bandage had gained particular significance in military and civilian settings alike [17]. Effective pressure dressing application requires achieving pressures sufficient for hemostasis without occluding arterial perfusion [16, 18–20]. 

Despite widespread inclusion in numerous civilian and military trauma training concepts, guidelines, and public hemorrhage-control initiatives, including PHTLS, TCCC (Tactical Combat Casualty Care), “Stop The Bleed”, WHO (World Health Organization), and ICRC (International Committee of the Red Cross) teaching materials, pressure dressing application lacks unform standards. A considerable heterogeneity exists regarding recommended materials and techniques, including circumferential wrapping, deliberate twisting, short pulling motions or pressure layers [13, 14, 21–31]. While current guidelines do include the pressure dressing, they do not provide precise specification regarding material selection or standardized technique [5, 15]. 

Overall, the available recommendations reveal a structural problem. While the pressure dressing itself is considered an established measure, internationally uniform implementation standards detailing technical aspects are lacking [13, 21]. This lack of standardization has measurable consequences in practice. Studies document considerable variability in achieved application pressures, with measured values often substantially below recommended target ranges [18, 19]. Subjective estimates of application pressure by emergency medical personnel are unreliable, showing no correlation with objectively measured values [32]. Even with standardized systems like the Israeli Bandage, significant variability in achieved pressures has been observed, with values ranging from very low and thus insufficient to potentially harmful levels over 250 mmHg, exceeding those associated with compression neuropathy [18, 33, 34]. 

The present study aimed to objectively assess pressures achieved by emergency medical personnel applying pressure dressings using commonly available materials. The primary objective was to analyze maximum achieved pressure and spatial pressure distribution patterns across defined application zones using a calibrated pressure sensor system. Secondary objectives included the systematic identification of influencing factors, specifically material type, application technique and provider experience. The findings intend to contribute to evidence-based standardization of hemorrhage control technique and inform optimization of training in the prehospital environment.

## Methods

We conducted a prospective, single-center, simulation-based observational study from August 2024 to January 2025 at the Emergency Department of a Level I Trauma Center in Germany. Ethics approval was obtained from the Ethics Committee of the Hannover Medical School (11337_BO_K_2024). Emergency Medical Personnel were approached after patient handover on-site and informed written consent was obtained from all participants. Data was collected anonymously.

A total of 124 participants were asked to apply, according to personal preferences, either a pressure dressing or an Israeli bandage, to the right, dominant, arm of a male volunteer of the study-team. Inclusion criteria were employment in the emergency medical services and a voluntary participation. Exclusion criteria were the presence of medical reasons that would have prevented performance. Participants were instructed to treat the small sensor area (described below) as an actively bleeding wound and to apply the pressure dressing as they would in a real prehospital emergency, according to their individual clinical routing. No specific target pressure or hemostatic endpoint was defined. The simulation did not provide clinical feedback such as visible bleeding cessation, and distal perfusion assessment was not part of the protocol.

Paramedics (“Notfallsanitäter”) represent in Germany the highest non-physician prehospital qualification level and require a standardized three-year vocational training program. Emergency Medical Technicians (EMTs) in the present study corresponded to the German “Rettungssanitäter” personnel, representing a shorter basic level prehospital qualification below paramedic level. Trainees were paramedic trainees, enrolled in the training program at the time of the study.

Available materials to select and combine according to personal preference included wound gauze, elastic fixation bandages (10 cm x 4 m, Raucolast, Fa Lohmann & Rauscher International GmbH & Co. KG), medium-stretch bandages (8 cm x 5 m and 12 cm x 5 m, Salva Last, Fa Fuhrmann GmbH), medical fixation tape and Israeli Bandages (The Emergency Bandage, Fa First Care Products Ltd.). Personal items were allowed to be used, if needed. Technique and material selection was based solely on individual preferences and prior experience.

**Fig. 1:**
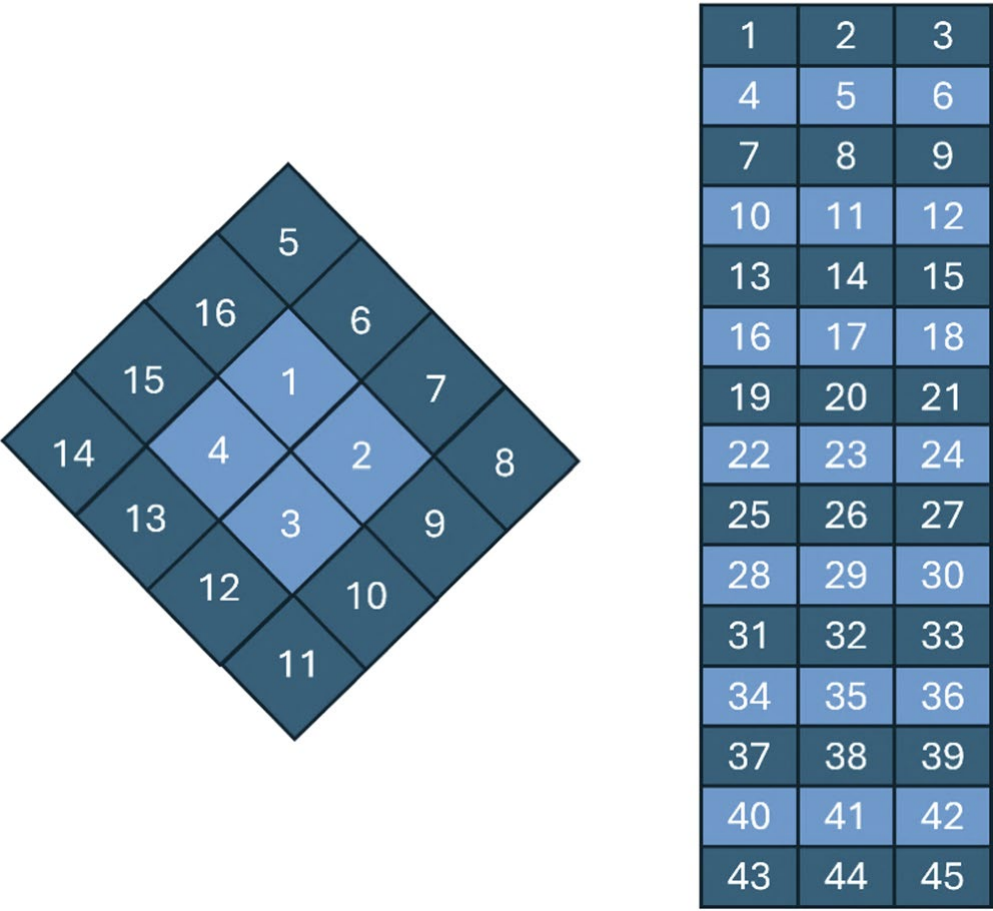
Distribution of measurements fields for used pressure sensors: Left: smaller sensor with 53 × 53 mm, 16 fields, positioned volar. Right: larger sensor with 175 × 46 mm, 45 fields, positioned circumferentially from dorsal

For pressure measurement a standardized set-up was used with two different capacitive pressure sensors (Fa Novel GmbH, Pliance^®^ System) with 16 and 45 measurement fields. See Fig. 1.

The smaller sensor (53 mm x 53 mm) was positioned on the volar forearm area and represented the predefined simulated wound area. The larger sensor (175 mm x 46 mm) was placed next to the smaller sensor and circumferential over approximately two-thirds of the forearm circumference. The larger sensor was used to capture spatial pressure distribution relative to the desired point of maximum pressure. The sensor array did not cover the full forearm circumference. Based on the forearm dimensions of the study volunteer a gap of approximately 21 mm remained on each lateral aspect between the volar and dorsal sensor, accounting for approximately 16% of the total circumference of the forearm. See Supplementary Data 1.

Before each measurement a calibration of the sensors was performed. Applied static pressure was recorded in kPa for each measurement field of both sensors and saved immediately after completion of bandage application via the Pliance^®^ Interface. The recorded pressure was subsequently converted to mmHg for reporting (conversion factor: 1 kPa = 7.501 mmHg), decimal values reflect this conversion rather than sub-unit measurement precision. The use of the same forearm was selected to enable reproducible sensor positioning and comparable pressure measurements under controlled conditions. The forearm length was measured with 290 mm and the circumference at the sensor placement with 270 mm.

In addition, application duration, material used, selected application technique, as well as gender, experience and professional background of participants were documented. The “short-tug technique” referred to intermittent short traction pulls applied during circumferential bandage wrapping to deliberately increase localized compression pressure, in contrast to continuous uniform wrapping tension. “Twisting” was defined as deliberate torsion of bandage material over the wound area during application. After completion of application participants were asked to rate their performance on a six-point ordinal scale from 1 (very good) to 6 (insufficient).

All data were compiled in Microsoft Excel (Version 2506 for Microsoft 365, Microsoft Corporation, Redmond, WA, USA). The simulation design focused on quantitative assessment of externally applied pressure distribution rather than direct physiological modeling of active hemorrhage.

Statistical analysis was performed using SPSS Statistics (V. 30, IBM Corporation, Armonk, NY, USA). For descriptive characterization of samples means, standard deviation (SD), medians, ranges and frequency distributions were calculated. Relationships between continuous variables were examined using either Pearson or Spearman correlations, depending on distribution assumptions. Group comparisons were conducted depending on data distribution either by ANOVA or non-parametric tests. For comparison of two independent groups Mann-Whitney-U test was used and for comparisons of more than two independent groups, the Kruskal-Wallis test. Where significant, exploratory pairwise Mann-Whitney-U comparisons were conducted without correction for multiple testing and findings were therefore interpreted descriptively. For categorical variables chi-square tests were applied. Comparison of pressure distribution between the volar and the dorsal forearm, as well as in the predefined cluster zones, was conducted based on zone-specific mean values. Further, a general linear model with repeated measures was employed. To identify independent predictors of maximum pressure of the volar sensor, multiple linear regression was also computed. Categorical predictors were incorporated into the model using indicator/dummy coding. Model assumptions were examined and results reported as regression coefficients with confidence intervals (CI). The significance level was set at α = 0.05.

## Results

A total of 124 participants completed the application of a pressure dressing. A total of 116 complete datasets were included in the final analysis of achieved pressures and pressure distribution, due to incomplete pressure sensor data in 8 cases. The majority of participants were male paramedics and emergency medical technicians (EMTs) with 0–5 years of experience. Most emergency physicians (EP) were trauma surgeons. *See* Table 1.

**Table 1:** Basic participant demographic and professional characteristics (*N* = 124)

**Sex**	***n*; % (100% =** ***N*** **124)**
Male	93; 75%
Female	31; 25%
Diverse	0; 0%
**Provider group**	**n; % (100% = N 124)**
Paramedics	45; 36%
Emergency medical technicians (EMTs)	54; 44%
Trainees	16; 13%
Emergency physicians	9; 7%
**Specialty of Emergency Physician**	**n; % (100% = N 9)**
Trauma surgery	6; 67%
Anesthesiology	2; 22%
Internal medicine	1; 11%
**Professional experience (in years)**	**6.12 ± SD 5.89 (0–25)** **n; % (100% = N 124)**
0–5	68; 55%
> 5–10	27; 22%
> 10	29; 23%

Trainees were included in the 0–5 category, based on the duration of their training at the time of the study

Out of the 124 participants 66% (*n* = 82) had previously applied a pressure dressing to a patient. The majority reported feeling confident in their skills during the application (78%, *n* = 97), while 22% (*n* = 27) did not. Confidence in performance showed descriptively a higher proportion of confident self-assessments among men (81.7%) than women (67.7%); however no statistically significant association was found (χ² (1) = 2.667; *p* = 0.102). Confidence was additionally influenced by the professional qualification and experience. 92.2% of those who have previously applied a pressure dressing in a real emergency situation reported feeling confident in the application, however this was true for only 55.3% of participants without “real-life” practical experience (χ² (1) = 23.316; *p* < 0.001). EPs (77.8%), paramedics (91.1%), and EMTs (75.9%) reported feeling confident, whereas only half of trainees (50%) felt confidence (χ² (3) = 12.039; *p* = 0.007). Participants with longer professional experience more frequently reported confidence (ρ = 0.381; *p* < 0.001).

Participants rated their application quality with a mean score of 2.17 ± 1.08 on a six-point scale with 1 being very good and 6 insufficient. Most participants rated their performance as “2; good” (57%, *n* = 71) or “3; satisfactory” (33%, *n* = 41), while only 3% (*n* = 4) rated their application as “1; very good”. Similarly, only few participants rated their performance as only “4; sufficient” (5%, *n* = 6) or “5 = poor” (2%, *n* = 2). No participant rated their application as “6 = insufficient”.

In a subgroup analysis, the median self-assessment after bandage application of male participants was “good”, while female participants rated their application with a median of “satisfactory”. However, this difference was not statistically significant (Mann-Whitney U test; U = 1192.0; *p* = 0.102; mean ranks: female = 70.55, male = 59.82); moreover, there was no significant difference in objectively measured maximum pressure (*p* = 0.449). The relationship between subjective satisfaction with the applied bandage and the actually achieved maximum pressure was examined using Spearman’s rank correlation. No significant correlation was found ρ = − 0.055 (*p* = 0.541). This thus indicates a lack of agreement between self-assessment and objective performance.

### Technique selection and pressure analysis

In almost all applications a sterile wound dressing before the bandage application was used (95%). The basic circumferential technique without twisting dominated in 75%, only 25% used twisting for increasing focal pressure. The majority of participants used short tugs for pressure maximization (82%). As the pressure layer/pad different materials were used, with the majority selecting one unwrapped bandage (87.1%) or two (7.3%). Other options were a self-made knot in the circumferential wrapping (3.2%) or a random hard object (2.4%). In 13% of applications the bandage was deliberately applied over a broader area > 10 cm. A pressure layer was used in 81%. Regarding material selection, a preference for elastic fixation bandages was seen, selected by approximately half of the participants (51%). A pressure layer/pad was used in 81% of cases. *See* Table 2.

**Table 2:** Selected materials and techniques for pressure dressing application (*n* = 124). Twisting = intentional twisting of the bandage to increase focal pressure

Wound dressing before bandage	*n*; % (100% = *N* 124)
Yes	118; 95%
No	6; 5%
**Wrapping technique**	**n; % (100% = N 124)**
Circular	93; 75%
Twisting	31; 25%
**Short-Tug application technique**	**n; % (100% = N 124)**
Yes	102; 82%
No	22; 18%
**Wide application (> 10 cm)**	**n; % (100% = N 124)**
Yes	16; 13%
No	108; 87%
**Pressure Layer**	**n; % (100% = N 124)**
Yes	103; 83%
No	21; 17%
**Bandage type**	**n; % (100% = N 124)**
Elastic conforming bandage	63; 51%
Medium-stretch bandage	36; 29%
Israeli bandage	25; 20%

Wide application = documented coverage > 10 cm of the dorsal forearm by the bandage; measured along the longitudinal axis of the forearm

Furthermore, analysis of material and technique selection by provider group revealed that physicians exclusively selected conventional bandages and applied the short-tug technique in all cases (100%). One third of paramedics and trainees selected the Israeli Bandage (31.1% and 31.2% respectively), EMTs chose the prefabricated system in 14.8%. Short-tug technique was employed by 80.0% of paramedics, 83.3% of EMTs and 75% of trainees.

The maximum recorded pressure on the volar sensor was 169.35 ± 84.33 mmHg, with a wide range of 50 to 412 mmHg. The mean duration of application was 51.20 ± 18.14 s, lasting in between 28 and 137 s.

### Professional experience

**Fig. 2:**
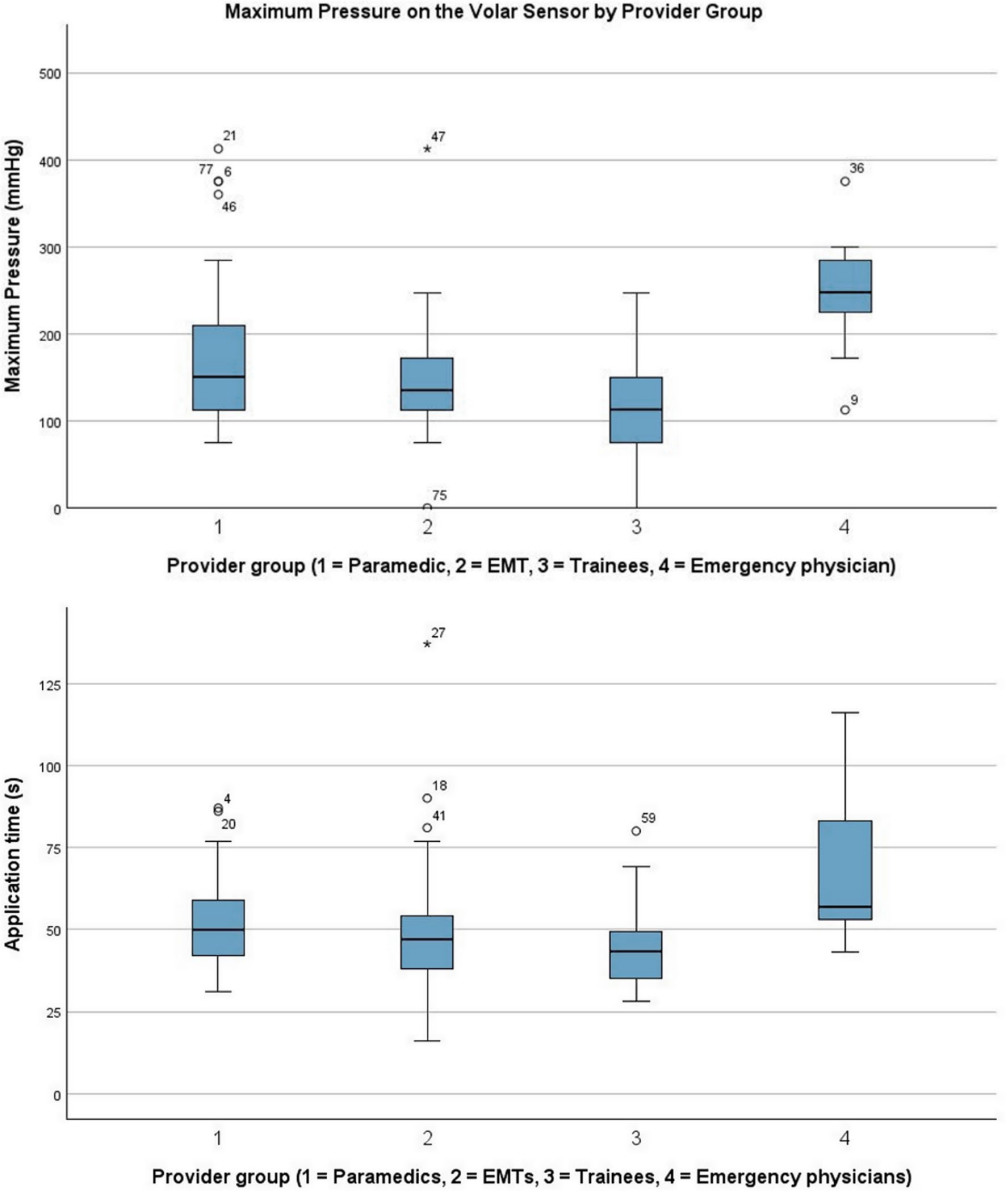
Influence of professional characteristics on maximum applied pressure and application time (Box plot, *N* = 116)

EPs generated higher mean maximum pressures than other participants. Within the group of paramedics, EMTs and trainees no statistically significant differences were found. Application duration varied moderately between professional groups. Emergency physicians applied bandages in a mean of 70.0 ± 27.2 s, paramedics in 52.0 ± 14.1 s, emergency medical technicians in 49.4 ± 18.8 s, and trainees in 44.6 ± 14.4 s. See Fig. 2.

A one-way analysis of variance revealed a significant difference between professional groups (F(3.120) = 7.18; *p* < 0.001, η² = 0.152; 95% CI [0.040; 0.254]). Due to violation of homogeneity of variance (Levene’s test: *p* = 0.012), Games-Howell post-hoc testing was performed, which confirmed significant differences between emergency physicians and all non-physician groups. The three non-physician professional groups did not differ significantly from each other. At the individual level years of professional experience also showed a significant positive correlation with maximum pressure achieved. The corresponding Pearson correlation was significant (*r* = 0.212; 95% CI [0.037; 0.374]; *p* = 0.018), suggesting a potential training effect or more intuitive handling among experienced personnel.

### Technique and materials

**Fig. 3:**
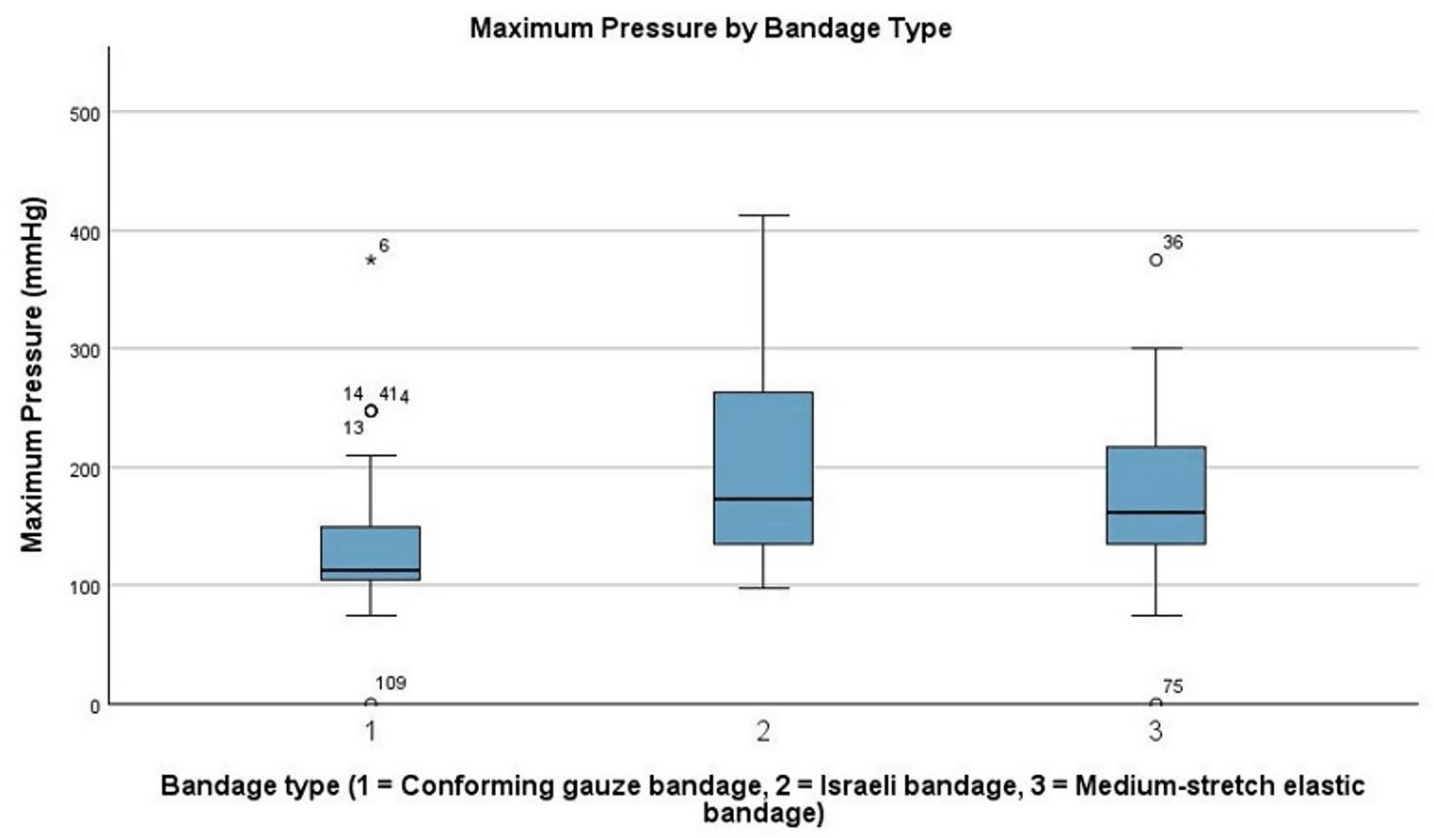
Influence of bandage type on maximum pressure of the volar sensor (Box plot, *N* = 116)

The selected bandage material showed a significant influence on the generated pressure. The highest pressures were observed with the usage of the Israeli Bandage (205.40 ± 98.12 mmHg), followed by medium-stretch bandages (171.68 ± 75.03 mmHg). Elastic fixation bandages (135.50 ± 58.63 mmHg) generated the lowest pressures. See Fig. 3.

Within the highest pressures a significant association was found between the selected bandage material and the occurrence of excessive pressure values > 250 mmHg. While only 1.6% achieved an excessive pressure using an elastic fixation bandage, a pressure over > 250 mmHg was recorded in 11.1% for medium-stretch bandages in 28% for the Israeli Bandage. The chi-square test confirmed this difference as highly significant (χ² (2) = 14.40; *p* < 0.001).

**Fig. 4:**
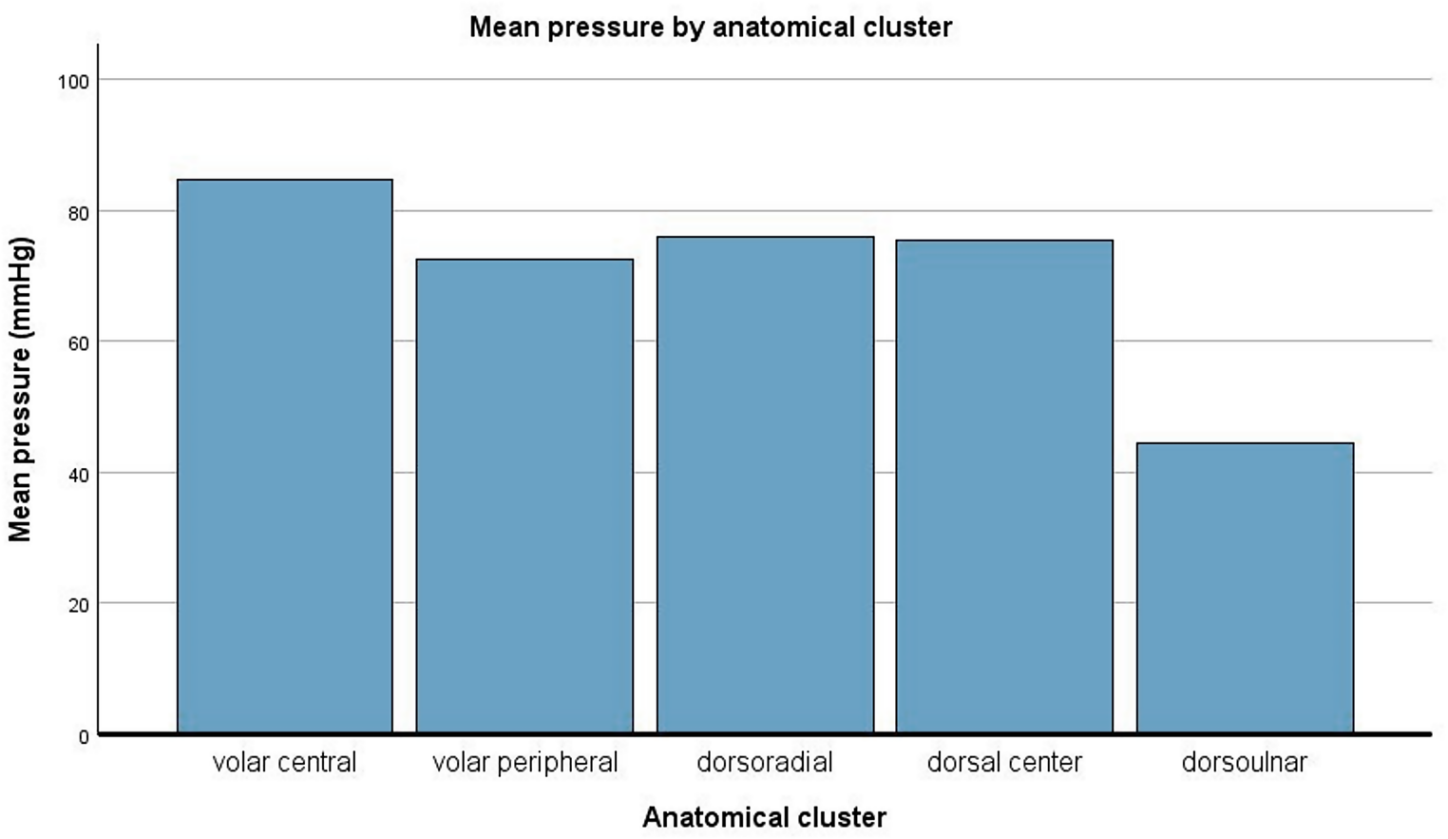
Pressure distribution (mean in mmHg) by defined anatomical clusters. *N* = 116

To examine whether the choice of bandage material affected local pressure distribution, a zone-specific analysis of mean area pressures was performed. The forearm was divided into five anatomically defined cluster zones; two volar areas and three dorsal zones. See Fig. 4 and See Table 3.

**Table 3:** Zone-specific pressure distribution by bandage type and short-tug application technique. *N* = 116

Anatomical zone	Mean ± SD (min-max) in mmHg	95%- CI of mean (mmHg)	Elastic conforming bandage (*n* = 63) Mean ± SD in mmHg	Israeli Bandage (*n* = 25) Mean ± SD in mmHg	Medium-stretch bandage (*n* = 36) Mean ± SD in mmHg	Effect of short-tug technique *r*; *p*-value
Volar central	84.81 ± 61.44 (0–292.52)	73.56–96.08	72.8 ± 58.1	97.8 ± 74.1	97.3 ± 55.8	*r* = 0.41; *p* < 0.001
Volar peripheral	72.47 ± 53.44 (0–234.39)	62.88–82.05	56.8 ± 44.7	89.9 ± 58.6	88.3 ± 57.1	*r* = 0.44; *p* < 0.001
Dorsoradial	76.11 ± 61.91 (0–243.35)	65.94–86.29	62.4 ± 53.8	103.2 ± 69.1	65.3 ± 65.3	*r* = 0.29; *p* = 0.002
Dorsal central	75.46 ± 78.40 (0–396.70)	61.02–89.91	58.5 ± 61.9	106.3 ± 110.1	74.8 ± 74.8	*r* = 0.30; *p* = 0.001
Dorsoulnar	44.42 ± 52.32 (0.00–201.68)	34.87–53.98	34.8 ± 45.9	69.0 ± 68.0	47.7 ± 47.7	*r* = 0.19; *p* = 0.040

**Fig. 5:**
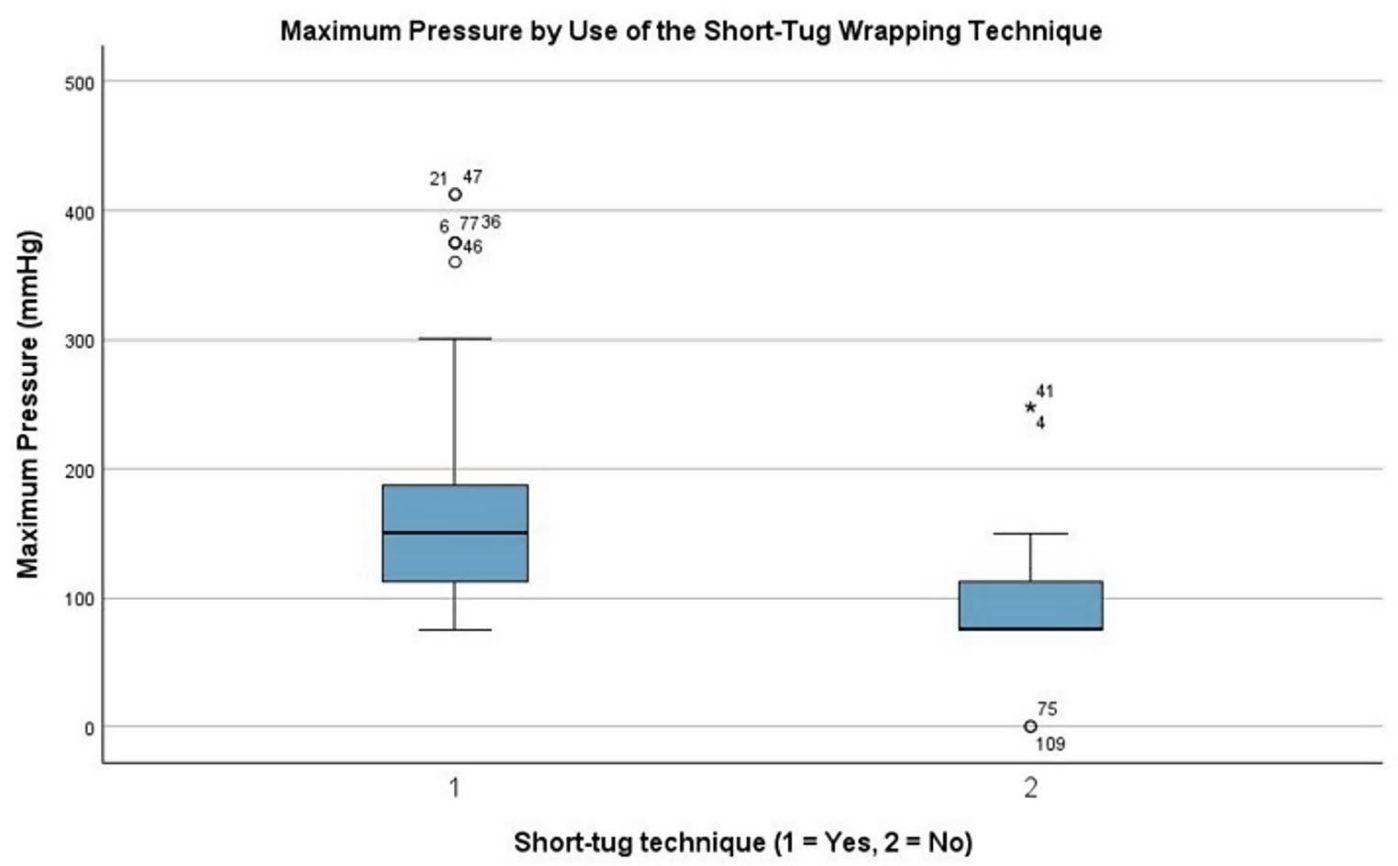
Influence of the short-tug technique on maximum pressure of the volar sensor (Box plot, *N* = 116)

An asymmetric pressure distribution was seen with significant differences between mean pressure values of the five zones (*p* < 0.001). Pairwise comparisons (Bonferroni-corrected) showed significantly higher pressures in the volar-central zone compared to the volar-peripheral area (*p* = 0.004) as well as compared to all dorsal zones, particularly in comparison to the dorsoulnar zone (*p* < 0.001). The other dorsal segments also showed significantly higher values compared to the dorsoulnar zone (all *p* < 0.001). Summarizing, the cluster analysis showed clear volar focusing with the highest achieved pressures in the central wound area, while the dorsoulnar zone consistently exhibited lower values. The wrapping technique also had a significant influence on the generated pressure. Particularly the use of short tugs was associated with higher maximum pressure over the volar sensor, as well as higher pressure of mean pressure values in five anatomically defined zones. Thus, the short-tug technique intensified this effect in almost all zones, without changing the general pressure pattern. See Fig. 5.

In addition, the dispersion was lower in the short-tug-group, indicating reproducible pressure transmission (Spearman rank correlation ρ = 0.452; *p* < 0.001; 95% CI [0.295; 0.585]; bootstrap sensitivity analysis with 1,000 resamples: 95% CI [0.255; 0.593]). A significant association was found between bandage material and the use of short tugs. While 73% of participants used short tugs with elastic fixation bandages, this proportion increased to 89% with medium-stretch bandages and 96% with the Israeli Bandage (χ² (2) = 8.007; *p* = 0.018). This suggests that material selection may influence technique choice.

A Kruskal-Wallis test showed significant differences between materials in the volar-central (*p* = 0.043), volar-peripheral (*p* = 0.008), and dorsoradial regions (*p* = 0.041), while differences in the dorsal-central and dorsoulnar regions were not significant (*p* = 0.190 and 0.083, respectively). Exploratory pairwise Mann-Whitney U comparisons revealed significantly higher pressures for medium-stretch bandages vs. elastic fixation bandages in the volar-central (*p* = 0.015; *r* ≈ 0.22) and volar-peripheral regions (*p* = 0.011; *r* ≈ 0.24). For the comparison between Israeli bandage vs. elastic fixation bandage, the Israeli bandage also showed higher pressures in the volar-peripheral (*p* = 0.013; *r* ≈ 0.23), dorsoradial (*p* = 0.014; *r* ≈ 0.23), and dorsoulnar regions (*p* = 0.032; *r* ≈ 0.20). No significant differences existed between the Israeli bandage and medium-stretch bandages (all *p* ≥ 0.20). Overall, no material-specific pressure peaks could be demonstrated on the dorsal surfaces.

To further examine pressure distribution between surfaces a general linear model with repeated measures was performed. Overall, higher mean pressures were measured on the volar surface (81.09 ± 5.17 mmHg; 95% CI [70.91; 91.23]) compared to the dorsal surface (69.66 ± 5.39 mmHg; 95% CI [58.99; 80.33]). The interaction effect Material × Surface was not significant (F(2.114) = 1.298; *p* = 0.277); thus the volar-dorsal difference did not differ statistically between materials. The main effect of material, however, was significant (F(2.114) = 5.786; *p* = 0.004; partial η² = 0.092): elastic fixation bandages achieved the lowest values, Israeli Bandage and medium-stretch bandages achieved significantly higher overall values (Bonferroni *p* = 0.009 for both comparisons), while Israeli Bandage and medium-stretch bandages did not differ significantly from each other (*p* = 1.000).

**Fig. 6:**
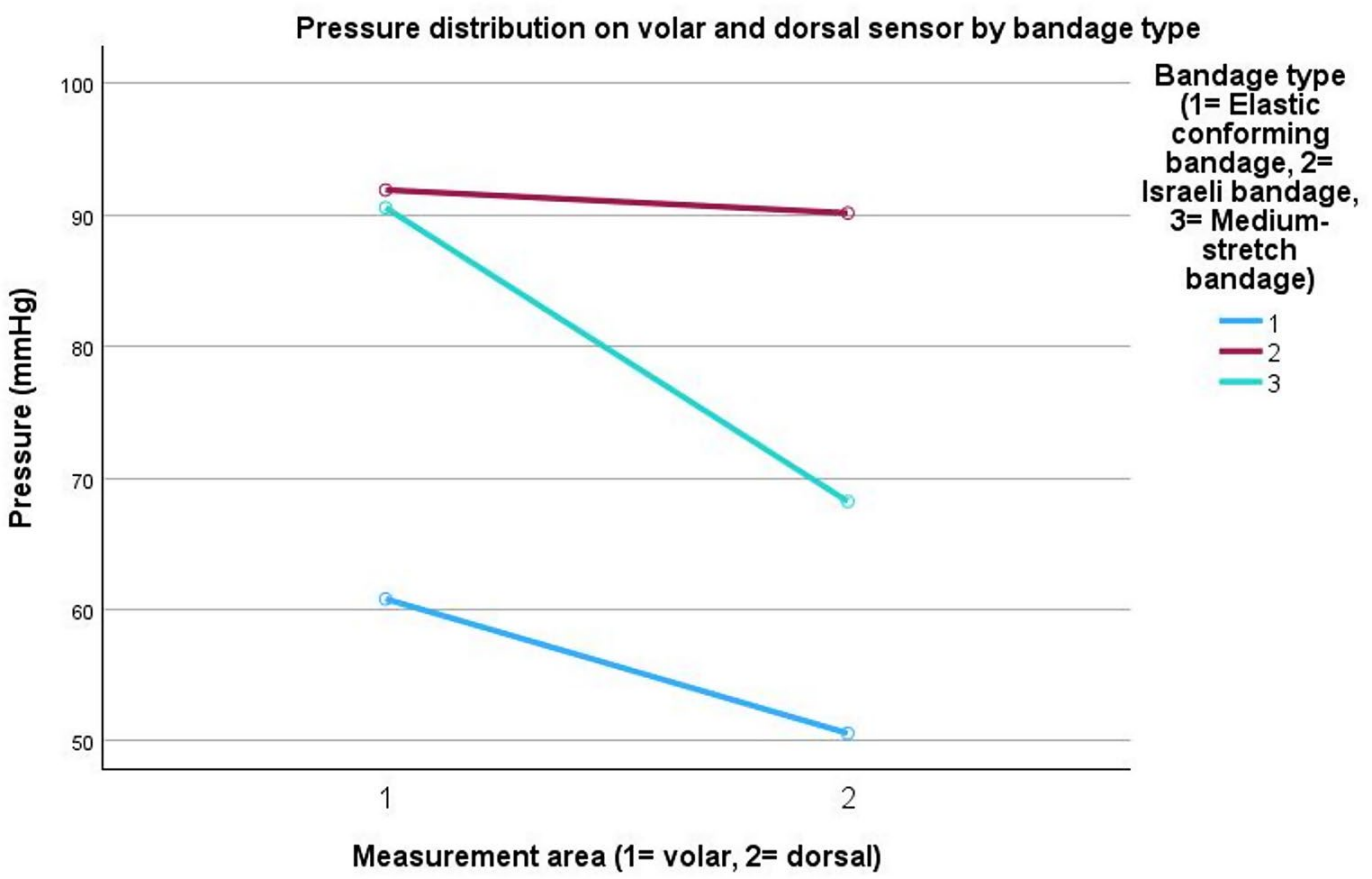
Mean pressure distribution of the volar and dorsal measurement area by bandage type. *N* = 116

Please See Fig. 6 for average pressure distribution as a function of bandage material used, illustrating a nearly balanced pressure distribution between volar and dorsal surfaces using the Israeli bandage. In contrast, with elastic or medium-stretch bandages a pressure distribution favoring the volar side is shown.

The usage of a pressure pad was analyzed for potential influence on pressure distribution between volar and dorsal surface. Four variants of a pressure pad were distinguished; mostly a package of simple gauze bandage was used. Alternatively, a knot in the bandage, a hard object or two overlapping gauze bandages was selected. No significant effect for the usage of a pressure pad was found (F(3.86) = 1.355; *p* = 0.262; partial η² = 0.045).

Deliberate twisting of the bandage showed no significant effect on achieved maximum pressure (χ² (1) = 0.06; *p* = 0.811). Furthermore, a significant positive association was found between application duration and maximum pressure generated. The Spearman rank correlation was ρ = 0.350; *p* < 0.001; 95% CI [0.180; 0.499] (bootstrap sensitivity analysis, 1,000 resamples: 95% CI [0.174; 0.491]), indicating a small to medium effect.

The influence of the wrapping width on pressure distribution using again a general linear model with repeated measures was performed. The main effect of wrapping width was not significant (F(1.115) = 0.028; *p* = 0.868), nor was the interaction between wrapping width and measurement surface (F(1.115) = 1.559; *p* = 0.214). Thus, no significant influence of wrapping width on pressure distribution between volar and dorsal sides could be demonstrated.

A multiple linear regression model was calculated to identify independent factors influencing the maximum pressure over the volar sensor. The model explained 19.9% of the variance (adjusted R² = 0.172; F(4.119) = 7.38; *p* < 0.001). The strongest predictor was the short tug technique. Material choice between conventional bandage and the prefabricated Israeli bandage also had a significant overall influence. Professional experience showed a slight positive effect, while gender had no significant influence. See Table 4.

**Table 4:** Multiple linear regression analysis for predicting maximum pressure. *N* = 116

Predictor	B (95%-CI)	*p*-value
Short-tug technique	64.2 (29.8–98.7)	< 0.001
Bandage type	16.1 (1.1–31.0)	0.036
Sex	–0.9 (–31.4–29.5)	0.952
Professional experience (years)	1.7 (0.07–3.3)	0.041

## Discussion

This prospective observational study analyzed pressure distribution of prehospital pressure dressing application by emergency medical service personnel. The objective was to quantify variability in achieved pressure values, identify influencing factors, and compare participants’ subjective assessments with objective measurements.

The substantial pressure variability documented in this study confirms findings from the only prior systematic investigation of pressure dressing application, where a significant proportion of a small sample size generated tourniquet-equivalent pressures risking compression injuries [18, 19]. Our data extend these findings by demonstrating, that almost 10% of aplications exceeded 250 mmHg, with material-specific risk profiles. This pressure threshold, derived from tourniquet and pressure dressing literature documenting nerve damage and ischemia risks with prolonged application [18, 33–36] provides a relevant upper safety reference.

Studies in venous compression therapy documented that material properties, wrapping technique and individual handling significantly influenced achieved pressures [37], factors we systematically quantified in the prehospital setting for the first time. This scarcity of evidence is notable, given that pressure generation through elastic bandaging has been extensively studied in other medical contexts, where a substantial proportion of compression bandages were applied with insufficient therapeutic pressure [20, 32, 37, 38]. 

Our results demonstrate that both professional qualification and longer practical experience measurably influence pressure dressing performance and higher achieved pressures. This is congruent with existing literature which shows that longer experience as well as frequent exposure in emergency medicine is linked to fewer errors, better accuracy and lower mortality [39–44]. 

However, emergency physicians exceeded the 250 mmHg threshold more frequently than other groups, suggesting that experience facilitates more forceful application, but not necessarily optimal pressure calibration. In addition, application duration also correlated with maximum pressure, with EPs requiring longer application times compared to e.g. trainees. Speculatively, this association may reflect more deliberate technique or greater attention to achieving adequate compression.

Confidence perception was strongly associated with practical experience. Participants with real-life application experience reported feeling more confident, this relationship parallels findings from “Stop The Bleed” training programs, where structured instruction and training significantly improved both application speed and quality [45]. 

Beyond experience levels, gender-specific differences in self-assessment following hemorrhage control training were observed [46, 47]. In our findings, women equally reported lower confidence and rated their performance more critically, though no significant differences existed in objectively measured maximum pressures. This pattern of systematic underestimation by women with equal objective performance has been documented across medical training contexts including hemorrhage control training [46–48]. Psychologically focused literature attributes such patterns to “motivated reasoning”, where expectations and self-images influence self-assessment independent of actual performance [49]. The observed lack of correlation between subjective assessment and objective pressure parallels findings from compression therapy, where personnel estimates proved similarly unreliable despite extensive experience [20, 32]. These findings highlight a critical training gap, as subjective feedback alone might be insufficient for assessment. Studies in compression therapy demonstrated that combining structured training with objective pressure feedback significantly improves application precision [38]. However, practical implementation remains challenging, as one-time training without reinforcement leads to rapid skill decay [50, 51]. Training programs must therefore address both technical skills and realistic self-assessment, particularly for groups prone to systematic underestimation, and incorporate regular refresher sessions with objective performance metrics.

Material selection and wrapping technique emerge as the strongest predictors of achieved pressure in the multiple regression model. However, it should be noted that the regression model explained a modest proportion of variance in maximum pressure, indicating that additional unmeasured factors likely contributed substantially to pressure generation. The identified predictors thus should be interpreted as associated factors rather than sufficient determinants of pressure generation. Among the evaluated techniques, the short-tug technique showed the most robust effect, consistently increasing pressure across all anatomical zones without altering the volar-focused distribution pattern. Its reproducibility may make it particularly suitable for standardized training protocols.

Several techniques showed no demonstrable benefit. Deliberate twisting of the bandage had no significant effect on maximum pressure. Broad bandage application > 10 cm and the use of a pressure layer showed no influence on pressure distribution. These negative findings suggest that training curricula should not emphasize these techniques.

Material selection was also significantly associated with pressure levels and risk profiles within the observational study setting. Applications using the Israeli bandage were associated with the highest recorded pressures, but exhibited a higher rate of excessive values, compared medium-stretch bandages. Notably, material selection also correlated with technique choice, as most of Israeli-Bandage users employed short tugs, suggesting potential confounding effects. Furthermore, physicians exclusively selected conventional bandages and universally applied the short-tug technique.

While no validated optimal pressure range specific for pressure bandages has been established, target ranges from adjacent literature provide a pragmatic clinical framework. For venous bleeding, pressures of 40–60 mmHg are typically considered sufficient, while arterial bleeding requires higher compression forces [16, 18–20]. The zone-specific pressure analysis revealed asymmetric distribution patterns with significantly higher values on the volar surface compared to the dorsal surface. This volar focusing with central pressure concentration corresponds to the therapeutic goal described in literature of targeted compression at the bleeding source while relieving opposing surfaces [17, 31, 52, 53]. Importantly, material choice did not significantly alter this fundamental distribution pattern, however, conventional elastic and medium-stretch bandages demonstrated a more pronounced volar predominance with comparatively lower dorsal pressures. This distribution approximates the therapeutically intended asymmetry much more closely. Israeli Bandages in contrast produced a more equalized volar-dorsal distribution reflecting a greater mechanical consistency, but not a more focused direct compression towards the volar bleeding source. In contrast to tourniquets, which intentionally achieve complete vascular occlusion, pressure dressings aim for hemostasis without distal ischemia. Unintended tourniquet-equivalent pressures represent application failure rather than therapeutic intent and may occur when visual or haptic feedback is absent [18, 19, 32–34, 37]. 

Interestingly, application duration (mean 51.20 ± 18.14 s) was comparable to tourniquet application times reported in training studies (30–77 s) [54–56]. This is relevant given the documented high rates of incorrect tourniquet application under operational conditions [36, 56]. A substantial proportion of casualties were documented to receive tourniquets for wounds where hemorrhage control could have been achieved by alternative means, and prolonged application time was associated with increased rates of amputations [57–59]. Pressure dressings which are applicable with equivalent speed using basic materials, may warrant further investigation as an alternative strategy for compressible hemorrhage. Systematic comparisons of tourniquets and alternative methods for hemorrhage control remain scarce, as do structured investigations of indication criteria [60–62]. This might represent an important area for future research also in disaster settings where resource constraints may necessitate prioritization of techniques with favorable side-effect profiles.

Several limitations warrant consideration. The monocentric design with possibly homogenous training background may limit generalizability. As material and technique selection were participant-dependent and non-randomized, it introduced potential self-selection bias with relevant co-selection effects between bandage type and application technique possibly influencing observed pressure differences. No a priory power calculation was performed. The relatively small subgroups limit statistical power for robust subgroup analyses. In particular, the emergency physician group was insufficiently powered. The observed pressure differences between Eps and non-physician groups, while statistically significant, should therefore be interpreted with caution and require replication in a larger sample. Hemostatic effectiveness was not directly verified, results relied on pressure values as surrogate markers for clinical efficacy. The standardized scenario enabled precise measurement, but did not reflect variables such as anatomical diversity, inaccessible bleeding sites or operational constraints including confined spaces, poor lighting, or dynamic rescue situations. The simulation design cannot replicate the dynamic conditions of real traumatic hemorrhage. In addition, the use of a study team member as the volunteer may have introduced social desirability bias. Despite these limitations, the combination of realistic data collection, precise sensor-based measurement, and systematic evaluation provides robust evidence for training optimization and standardization efforts.

## Conclusion

This investigation provides the first comprehensive quantitative analysis of pressure distribution in prehospitally applied pressure dressings. The identified variability in pressure application and material specific risk profiles necessitate concrete modifications to training and clinical practice.

For clinical practice and training, several concrete recommendations emerge: Technique instruction should prioritize the short-tug technique. Techniques without demonstrable benefit, such as deliberate bandage twisting or specific pressure pad configurations should not be emphasized in training curricula. Further, material selection requires context-dependent consideration. Prefabricated systems like the Israeli Bandage may offer advantages in time-critical or tactical settings but require targeted training to mitigate the risk of excessive pressure. In routine civilian care, classic pressure dressings, with medium-stretch bandages if available, remain appropriate first-line interventions. Clinical monitoring after application is essential with systematic assessment of distal perfusion, including skin-color, capillary refill time and neurovascular status.

Training programs should incorporate simulation-based learning with objective feedback mechanisms and regular refresher training. Programs must address both technical execution and clinical assessment competencies, with particular attention to groups prone to systematic performance underestimation. These findings may be relevant for standardized civilian and military training concepts, including PHTLS and TCCC, where rapid and reproducible management of compressible hemorrhage represents a core principle.

Further research should evaluate hemostatic effectiveness under variable operational conditions. Given emerging evidence of tourniquet overuse and associated morbidity in recent armed conflicts, comparative studies examining indication criteria and outcomes for pressure dressings versus tourniquets, particularly in mass casualty and prolonged-care scenarios, should inform civilian trauma, military and disaster medicine protocols.

## Supplementary Information

Below is the link to the electronic supplementary material.

Supplementary Material 1

## Data Availability

The datasets used and/or analyzed during the current study are available from the corresponding author on reasonable request.
