# Supplementary material for: Under pressure - a sensor-based analysis of simulated prehospital pressure dressing application for hemorrhage management
Source: Eur J Trauma Emerg Surg. 2026 Jun 15;52(1):192. doi: 10.1007/s00068-026-03247-9 (PMC13269437; doi:10.1007/s00068-026-03247-9)
Supplement: Supplementary file 1 — Supplementary Material 1 [file 68_2026_3247_MOESM1_ESM.docx]

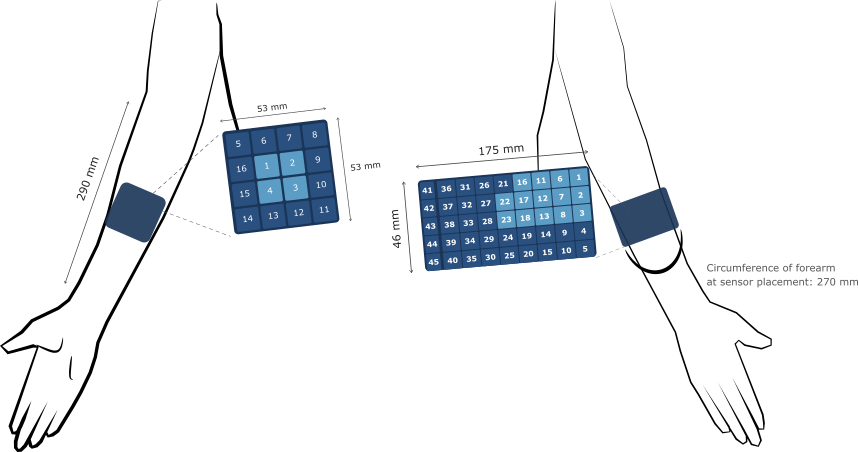


Supplementary Data 1. *Schematic illustration of the experimental set-up. Right forearm of a male volunteer, dominantly used hand. Forearm length: 290 mm. Circumference at sensor placement: 270 mm. Volar sensor placement of the 53x53mm sensor and dorsal placement of the 175x46 mm sensor.* Illustration done with Inkspace: Open Source Scalable Vector Graphics Editor V. 1.4.4 for macOS (licensed under GNU general public license V2.0)
